# Disease Models & Mechanisms in the Age of Big Data

**DOI:** 10.1242/dmm.041699

**Published:** 2019-08-20

**Authors:** Antonis K. Hatzopoulos

**Affiliations:** Department of Medicine, Division of Cardiovascular Medicine, Vanderbilt University Medical Center, Nashville, TN 37232, USA

**Keywords:** Big Data, Disease modeling, GWAS, Omics

## Abstract

In the decade since Disease Models & Mechanisms was launched, the emergence of Big Data as the main foundation of biological information is having a profound effect on how we do research and it has provoked some interesting questions. Is Big Data exploration replacing hypothesis-driven basic research? And, to what extent is disease modeling in the laboratory still relevant to medical research? Recent examples of synergistic approaches utilizing animal modeling and electronic medical records mining show that combining efforts between disease models and clinical datasets can uncover not only disease etiologies, but also novel molecular and cellular mechanisms linked to gene function.

## Abstract (precis)

**Summary:** This Editorial reflects on how the emergence of Big Data has affected traditional disease modeling over the past decade, and on DMM's place in this changing landscape.

## Introduction

In a series of editorial articles in the inaugural issue of Disease Models & Mechanisms (DMM), Vivian Siegel, the first Editor-in-Chief, and members of The Company of Biologists and the Editorial Board laid out how the idea for the new journal was conceived, outlined its scope and expressed their hopes and expectations for the new venture (Siegel, 2008; Freeman and St Johnston, 2008). They envisioned the launch of the new publication as a forum to foster and broaden the interactions between what they considered as two poorly connected scientific silos, biological research and animal models in one, and clinical research and medicine in the other. Much has changed since the start of DMM over ten years ago, for the relationship between biological and clinical research has greatly evolved. Although this partnership faces new challenges, as the volume, complexity and heterogeneity of scientific information continues to expand, it also promises to revolutionize both basic biological knowledge and medical care. Part of this revolution stems from the emergence of Big Data as a centerpiece of scientific discovery, offering an unprecedented opportunity to determine biological and disease mechanisms (Schüssler-Fiorenza Rose et al., 2019).

Big Data come in many different forms, for example whole-genome sequencing, RNA sequencing-based transcriptomics (bulk and single cell), proteomics, metabolomics, Genome-Wide and Phenome-Wide Association Studies (GWAS, PheWAS), and Electronic Medical or Health Records (EMR/EHR). Particularly exciting as discovery tools are EHRs that contain extensive phenotype information, which is accompanied by DNA and/or tissue biobanking and genomic information, such as the Vanderbilt University Medical Center BioVU database, the Electronic Medical Records and Genomics (eMERGE) Network, or the United Kingdom (UK) Biobank (Roden et al., 2008; Gottesman et al., 2013; Sudlow et al., 2015).

The collection, storage and mining of increasingly diverse and complex types of biological information from the general human population, or from patients with well-documented medical records – based on diagnoses organized by ICD9/10 codes and accompanied by laboratory test results, imaging and other procedures – is having a profound effect on modern-day research. This enormous data collection has thrusted humans, as the ultimate experimental model, to not only understand the pathophysiology of different diseases and to develop new therapeutic approaches, but also, rather unexpectedly, to understand basic gene function and biological mechanisms.

This emerging landscape has brought new challenges in the communication and translation of information among different disciplines, demands for new skills, and a need for innovative and sophisticated research tools. It seems that the separate research silos, which the DMM founders saw as a major impediment ten years ago, have been swept away into a vast ocean of information and Big Data, an ocean we must all learn to swim in. We can now sequence all variants in a human genome from all patients and relate them to RNA and protein levels, metabolites, or functional and phenotypic traits. Yet, how to parse, analyze and, most importantly, extract meaningful information from these datasets is not always straightforward. It is evident that Big Data depend on basic biology to understand disease mechanisms. Equally, biology needs Big Data to understand the pleiotropic effects of gene function and generate knowledge that is relevant and consequential in the current scientific climate.

## Why Big Data needs biology

In the last decade, GWAS have identified numerous disease-associated loci (Visscher et al., 2017). Yet, using these links to determine disease etiology or mechanisms can be challenging. In many instances, it is hard to identify the candidate gene using GWAS because single-nucleotide polymorphisms (SNPs) are frequently found outside coding sequences in genomic regions surrounded by many genes. Current experience indicates that the closest to the SNP gene is not always the disease-causing one (Smemo et al., 2014). Another limitation may stem from the fact that GWAS typically search for specific phenotypes, potentially missing additional clinical features that share the same genetic cause. Therefore, often one has to know where and how to search in order to untangle disease-causing mechanisms, especially in complex diseases. Biological evidence in animal models may help shape and guide these searches to pinpoint the gene responsible for the phenotype, reveal the full extent of gene pathogenicity, and provide functional validation and mechanistic insights in animal models using gain- or loss-of-function approaches (Unlu et al., 2019a).

From a practical point of view, GWAS and other Big Data investigations that are based on statistical associations rely on Bonferroni corrections or similar methods to improve confidence in the results, often requiring extremely low *P* values to accept that an association is real. Although such strict requirements are necessary to accept statistical associations, they can also cause researchers to discard many findings, especially if the relevant cohorts are relatively small, for example in studies aiming to establish the pathogenicity of rare variants (Bastarache et al., 2018). Biological evidence in an appropriate experimental model can provide independent validation of the findings, lowering the high statistical thresholds for acceptance and opening the doors of discovery (Unlu et al., 2019).

## Why biology needs Big Data

Although it might be easier to imagine why gene-disease phenotype associations, obtained by statistical means, need wet lab experimental support for validation and mechanistic insights, it is often less obvious how wet lab biology can benefit from Big Data, especially EHRs. Although the use of clinical data to assess the putative significance in human disease of a mechanism that was discovered in an animal model is expected, analyzing clinical records also has the potential to reveal new aspects of gene function that were not picked up in the laboratory.

The ‘biology of real life’ is more diverse and complex than ‘biology in the laboratory’. Animal research is often performed in genetically identical animals (an exception is outbred zebrafish), yielding results within the context of specific gene-gene interactions, and under communal environmental conditions, thus obscuring the pathogenicity of gene malfunction in a population-at-large setting. Although such strictly controlled parameters have traditionally been necessary to perform fundamental research studies, crucial aspects in a disease pathology may also include environmental influences, behavioral patterns, lifestyle, diet, interactions with microbiota or cultural influences, each dictating to various degrees to what extent aberrant gene function contributes to disease initiation, progression or severity. These aspects cannot be easily recapitulated in the laboratory and, therefore, laboratory analyses, no matter how sophisticated, may fail to capture the full phenotypic gamut and pathogenic potential of some genes. On the other hand, these features may be uncovered in large cohorts with clinical or epidemiological data.

Most of the described phenotypes in animal models are usually based on loss-of-gene-function approaches. In human subpopulations, mutations or variants can also be gain-of-function, or exert dominant-negative effects, interfering with the activities of an entire gene family or disrupting the proper function of complex genetic networks; or, certain variants might represent partial loss of function or cause variance in gene expression in specific tissues (Müller et al., 2013; Gamazon et al., 2018; Li et al., 2019). If some of these variants cause relatively mild perturbations in gene activity they likely remain benign, except in cases when the system is stressed, for example by injury, environmental factors or a co-occurring disease like diabetes. Also, most studies in a variety of injury models are more often than not performed in young and otherwise healthy animals without comorbidities. And, although things are gradually improving thanks to current NIH guidelines, most work in the past has been carried out in male animals. Conversely, the effects of sex-differences, aging or comorbidities to disease-contributing genetic alterations might be embedded in large EHR datasets, providing a more comprehensive and complete image of gene function.

Genetic animal models are usually based on alterations in the expression and/or activity levels of a single gene. On the other hand, diseases are often the result of defects in many molecular or cellular components. Furthermore, certain genes, besides generating a recognizable phenotype in a specific organ, might also be associated with a behavioral trait or mood disorder that would likely be missed in fish or mouse models, an association that could be documented in clinical datasets. As a result, the full spectrum of clinically relevant phenotypes associated with malfunction of a gene product may not be captured in an animal model, or they may be simply overlooked because particular phenotypes are not recognized. Adding to that, as information of single-cell RNA-seq data accumulates, it appears that apparently similar cells exhibit functional and phenotypic heterogeneity that may not be evident in a controlled experimental setting, but it may be revealed in a complex population setting. It is likely some of these features will be uncovered in large human-population datasets and EHRs, which offer enormous sample sizes that are not feasible in the laboratory. Newly discovered features in humans could be then further probed for pathomechanisms, with additional tests in available animal models, or guide the generation of new models more suitable to address a specific issue.

Other important aspects of gene (mal)function that may be missed in the laboratory but can be detected in clinical datasets include long-term effects that will not manifest in animal models with short life or observational time spans. On the other hand, analyses in EHR or epidemiological studies can provide longitudinal data to complement or expand animal studies (Schüssler-Fiorenza Rose et al., 2019).

## Exploring synergies

Recent elegant studies highlight the tremendous value of exploring synergies between Big Data in EHRs and biological research, especially with the advent of technologies such as CRISPR-Cas9 that have dramatically enhanced the fidelity, precision and efficacy of introducing gene variants into animal models. In one example, using BioVU and other databanks, investigators used EHR-linked genetic samples to discover gene-disease associations that were then validated in zebrafish loss-of-function models. Analysis of mutant zebrafish revealed additional phenotypes that were then verified in human patients by targeted searches among clinical phenotypes (Unlu et al., 2019). In another notable study, whole-exome sequencing in a patient with a serious lymphatic anomaly revealed a variant in *ARAF*, a gene encoding a kinase that acts upstream of the MEK/ERK signaling cascade. Modeling of the variant in human cells and zebrafish revealed overactive MEK/ERK signaling causing abnormal lymphatic morphology. The lymphatic abnormality was rescued in zebrafish with available MEK inhibitors. Treatment of the patient with a MEK inhibitor led to remodeling of the patient's lymphatic system and a remarkable improvement in his medical condition and quality of life (Li et al., 2019).

It appears that we are entering a powerful new symbiotic relationship in which Big Data depend on biological model systems to validate statistical associations, and basic biological research needs Big Data to capture the complexity of gene function outside the well-controlled environment and genetics of the laboratory. Although Big Data cannot replace hypothesis-driven basic biological studies in animal models, taking some of the early success stories into account, it is evident that better integrating the two areas of disease models and Big Data from EHRs, at the technical and personnel level, will uncover disease mechanisms and draw us nearer to fulfilling the expectations of personalized medicine. DMM is poised to play a vital role in accomplishing this goal by nurturing the emerging synergies between clinical and biological research. Off to the next decade!
